# Nuclear Envelope Integrity in Health and Disease: Consequences on Genome Instability and Inflammation

**DOI:** 10.3390/ijms22147281

**Published:** 2021-07-06

**Authors:** Benoit R. Gauthier, Valentine Comaills

**Affiliations:** 1 Andalusian Center for Molecular Biology and Regenerative Medicine-CABIMER, Junta de Andalucía-University of Pablo de Olavide-University of Seville-CSIC, 41092 Seville, Spain; 2 Centro de Investigación Biomédica en Red de Diabetes y Enfermedades Metabólicas Asociadas (CIBERDEM), 28029 Madrid, Spain

**Keywords:** nuclear envelope, nuclear envelope disruption, inflammation, cGAS/STING, chromosomal instability, envelopathy, cancer, lipodystrophy, neuropathy

## Abstract

The dynamic nature of the nuclear envelope (NE) is often underestimated. The NE protects, regulates, and organizes the eukaryote genome and adapts to epigenetic changes and to its environment. The NE morphology is characterized by a wide range of diversity and abnormality such as invagination and blebbing, and it is a diagnostic factor for pathologies such as cancer. Recently, the micronuclei, a small nucleus that contains a full chromosome or a fragment thereof, has gained much attention. The NE of micronuclei is prone to collapse, leading to DNA release into the cytoplasm with consequences ranging from the activation of the cGAS/STING pathway, an innate immune response, to the creation of chromosomal instability. The discovery of those mechanisms has revolutionized the understanding of some inflammation-related diseases and the origin of complex chromosomal rearrangements, as observed during the initiation of tumorigenesis. Herein, we will highlight the complexity of the NE biology and discuss the clinical symptoms observed in NE-related diseases. The interplay between innate immunity, genomic instability, and nuclear envelope leakage could be a major focus in future years to explain a wide range of diseases and could lead to new classes of therapeutics.

## 1. Nuclear Envelope Biology

**Figure 1:**
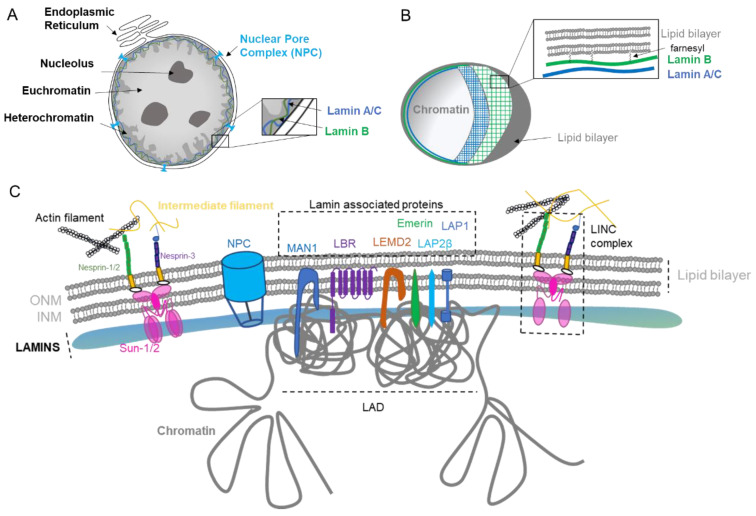
Nuclear envelope composition and organization. (**A**). The nucleus is surrounded by the nuclear envelope (NE). The Outer Nuclear Membrane (ONM) is continuous with the endoplasmic reticulum. The nuclear pore complex (NPC) regulates the export and import between the nucleus and the cytoplasm. The genome is organized in different compartments: euchromatin, heterochromatin, and nucleolus. (**B**). Structure of lamin layers in the Inner Nuclear Membrane (INM). (**C**). The NE is composed of a lipid bilayer anchored by several proteins forming the lamin-associated protein, the LINC complex, and by the lamins. The NE proteins regulate gene organization with the Lamin-Associated Domain (LAD).

The main role of the nuclear envelope (NE) is to compartmentalize and protect the unfolded genomic DNA from the cytoplasm in eukaryote cells. It is composed of a lipid bilayer reinforced in its inner side with a sheet-like structure of proteins called the nuclear lamina. The outer nuclear membrane (ONM) shares a common border with the endoplasmic reticulum (ER). The nuclear envelope has also many nuclear pores that facilitate the transport of molecules between the cytosol and the nucleus (Figure 1A). The NE is a dynamic organelle that expends, disrupts and reconstitutes during mitosis. In the interphase, it constantly remodels to adapt to nuclear growth.

### 1.1. Nuclear Lamina

The lamina conveys strength, flexibility, and rigidity as a function of its variable composition and ratio among the various lamins [1,2]. In addition to providing mechanical support and being an anchorage platform, the nuclear lamina regulates important cellular events such as DNA replication [3] and cell division [4]. The lamina is primordial for gene regulation [5,6] through the repression of genes in the Lamin-Associated Domain (LAD, Figure C), DNA repair [7], organization of the nucleolus [8], as well as chromosomal positioning [9]. The Lamina contributes to the organization of the genome into its different compartments: (i) the heterochromatin, characterized by repressed DNA that is tethered into the LAD, (ii) the euchromatin, the active compartment with a loose chromatin structure that is active for transcription, and (iii) the nucleolus, the site of ribosome production and assembly (Figure 1A).

The lamina also possesses mechano-responsivity in order to adapt to the cell’s environment. Lamin levels are dynamic, regulated by cell differentiation, and depend on the tissue stiffness [2,10]. Lamins are interconnected to the cytoskeleton by intermediate proteins such as the Linker of the Nucleoskeleton and Cytoskeleton (LINC) complex composed by SUN1/2 and Nesprins proteins (Figure 1C), which allow sensing and rapid cellular response via the complex post-translational control of its proteins [2,11]. The NE can also adapt in response to stiffness [2] or to function. For example, migrasive and invasive cells need to acquire nuclear elasticity and can decrease nuclear envelope proteins in order to compress through narrow space [1,10,12].

The nuclear lamina is composed of lamins and nuclear lamin-associated membrane proteins (Figure 1B,C). Lamins are type V intermediate filaments fibrous proteins that are divided into two major categories, the A and B type. The gene *LMNA* can be spliced in two predominant isoforms, the longer version encoding the protein Lamin A and the shorter isoform generating the Lamin C protein. The type B lamins are expressed by two different genes: *LMNB1* and *LMNB2*, encoding Lamin B1 and B2, respectively. While Lamin A is expressed in differentiated cells, type B lamins are ubiquitously expressed in all cells. Similar to other intermediate filament proteins, lamins self-assemble into complex structures. Lamins are highly dynamic and regulated proteins that assemble and disassemble pending stimuli [13]. They are organized into distinct networks at the nuclear periphery [14] (Figure 1B). Lamin B1 forms an outer concentric ring, and its localization is curvature-dependent. This suggests a role of Lamin B1 in stabilizing nuclear shape by restraining outward protrusions of the Lamin A/C network [15]. Lamins are subject to numerous post-translational modifications, most prominently phosphorylation, such as Lamin A that harbors more than 70 identified unique phosphorylation sites. Such phosphorylation regulates/coordinates the different structural state of lamins. For example, during interphase, Lamin A phosphorylation on Serine S22 and S390 promotes lamin degradation and nuclear softening in response to low cytoskeleton tension [16,17]. Other post-translational modifications include farnesylation, sumoylation, and acetylation [18]. Of particular interest is the farnesylation of the carboxyl terminal end cysteine (CaaX) that anchors lamins into the lipid layers. Type B lamins are permanently farnesylated (Figure 1B), while type A lamins are only transiently farnesylated before the carboxy-terminal peptide is released by the cleavage of prelamin A by ZMPSTE24 to form the mature Lamin A protein. Lamin C proteins are not farnesylated due to their lack of the CaaX motif. Interestingly, the majority of premature aging diseases such as the Hutchinson–Gilford progeria syndrome aka progeria ensue from single point mutations within the *LMNA* gene that give rise to a permanently farnesylated mutant Lamin A protein, which is termed progerin.

Other important constituents of the lamina are the lamin-associated proteins that mediate the attachment of lamins to the nuclear envelope. Their role is to assist lamins in regulating the chromatin, as exemplified by Lamin B Receptor (LBR) that interacts with several histone modifiers [19], as well as facilitating mechanotransduction as for Emerin [20] and regulating signaling pathways such as TGFβ by MAN1 [21] (Figure 1C). The diversity of lamin-associated proteins justifies the various roles and functions played by the nuclear lamina.

### 1.2. Nuclear Pore Complex

The nuclear pore complex (NPC) is one of the largest macromolecular assemblies in cells [22] composed of approximately 1000 protein subunits, which are named nucleoporins (NUP). The main function of the NPC is to control the trafficking in and out of the nucleus by allowing the passage through the lipid layers (Figure 1A,C). Moreover, NPC is known to have important functions in chromosomal organization and gene regulation, as it can interact with the genomic region enhancers and super enhancers [23].

### 1.3. Nuclear Lipid Bilayer

The nuclear envelope (NE) is composed of two phospholipid bilayers organized in an inner nuclear membrane (INM) and outer nuclear membrane (ONM) separated by a lumenal space (Figure 1B,C). The layers are composed of several kind of lipids with different physical properties such as cylindrical lipids (phosphatidylcholine, PC) and conical lipids (phosphatidylethanolamine, PE, diacylglycerol, DAG). Eukaryotic cells maintain their membrane lipid composition within narrow limits with phosphatidylcholine (PC) being the most abundant phospholipid in their nuclear envelope [24]. Despite this relative stable composition, studies in yeasts have shown that pending the environment, de novo specific lipid synthesis can take place in order to relieve the curvation elastic stress on the nuclear membrane protecting the nucleus from breakdown [24]. Interestingly, PCYT1A, the rate-limiting enzyme of PC synthesis involved in this curvation compliance, is localized at the INM in mammalian cells, suggesting a similar adaptability in response to stress and cellular needs in higher organisms [24]. Interestingly, it has also been shown that yeast cells keep lipid droplets at the INM, highlighting that INM has its own lipid metabolism and striking metabolic adaptability [25]. The formation of those lipid droplets through nucleation is influenced by membrane proteins, lipids, and mechanical properties [26]. Thus, multiple adaptability mechanisms may exist to selectively enrich and regulate specific lipid species at the INM in eukaryote cells [27].

## 2. Nuclear Envelope Diversity and Abnormalities

**Figure 2:**
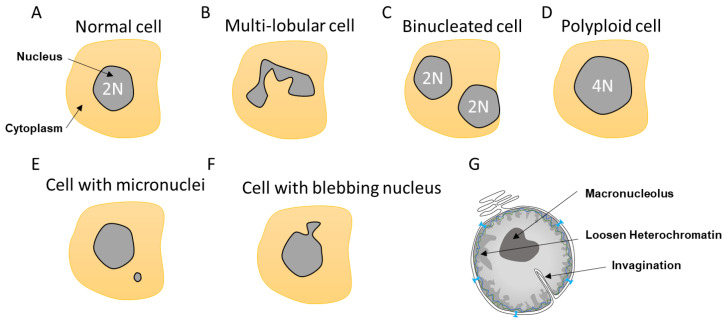
Different nuclear abnormalities. (**A**) Classical cells have one single round nucleus that contains a diploid genome (2N). (**B**) Cells such as neutrophils possess multi-lobar nucleus. (**C**,**D**) Polyploid cells (>4N) can either have two or more separated nuclei or one enlarged nucleus. (**E**) Some cells can have a smaller nucleus, which is called a micronucleus, in their cytoplasm. Micronuclei contain a full chromosome or a piece thereof. (**F**) Nuclei can also display blebbing characterized by an outward extension of the nuclear envelope (NE). (**G**). In addition, cancer cells can show abnormalities such as NE invagination, loosen heterochromatin as a consequence in changes in DNA compaction as well as a single and bigger nucleolus, which is called a macronucleolus.

Nuclear morphology is a common marker for cell determination and classification [28,29]. The nucleus can transpire defects depending of its cell state, aging, or upon mutations. Instead of the typical circular shape, the nucleus can be enlarged, loose circularity, displaying invaginations and a macronucleolus. It can also suffer from altered heterochromatin and irregular nuclear margins as well as a loss of proper compartmentalization involving NE collapse or leakage (Figure 2).

### 2.1. Multi-Lobular Nucleus, Micronuclei, Macronucleoli, and Invagination

Most cells contain a single circular nucleus with one copy of the genome (Figure 2A). However, depending on the nuclear envelope flexibility and abnormalities, the nuclear shape can vary. Some cells have a multi-lobular nucleus such as the neutrophils that required special flexibility needs due to their invasion properties (Figure 2B). Other cells can be polyploid due to an extra copy of the genome sequestered either in a separated nucleus, as in the case of binucleated cells, or contained within the same nucleus, generating an enlarged nucleus (Figure 2C,D). These polyploid cells can arise by a variety of mechanisms, including mitotic slippage, cytokinesis failure, endoreplication, and viral-induced cell fusion [30,31]. Polyploid cells can be found in the pancreas, placenta, muscle, lactating breast, liver, or heart tissues to either improve cell function or support tissue repair and regeneration [31]. Such normal polyploid cells are strictly controlled and not proliferative and are likely senescent. However, it was described in the case of liver that senescence could be reversible and hectaploid hepatocytes may re-enter mitosis, giving rise to tetraploid and diploid cells, which is a dynamic process referred to as a ‘ploidy conveyor’, highlighting the mitosis plasticity of such cells [31,32].

Other nuclear abnormalities include the formation of small nuclei in close proximity to the nucleus (Figure 2E). These small nuclei, referred to as micronuclei (MN), contain either an intact chromosome or pieces thereof and stem from inappropriate chromosome segregation during the anaphase. This DNA, which is excluded from the main nucleus, will attract NE components to form an MN. These extra nuclei bodies have in recent years come into the limelight, as they are a source of substantial DNA damage and can generate diverse complex chromosome rearrangements that are observed in several diseases such as cancer [33].

Nuclear morphology can also be severely altered as a result of impaired nuclear rigidity precipitated by either disruption of the lamina content and chromatin compaction [34] or to increased cytoskeleton forces during attraction and invasion [10,35,36] causing NE blebbing (Figure 2F). This lack of NE strength can also lead to the formation of invaginations, which can affect both the inner and outer nuclear membrane [37]. Invaginations can enclose cytoskeletal elements, both actin and cytokeratin intermediate filaments [38] (Figure 2G). In view of the intimate connection between epigenetic modifiers and the lamina, it is not surprising that both blebbing and MN provoke alterations in the epigenetic landscape [17].

### 2.2. Apoptosis, Senescence, and Aging

The nuclear envelope integrity can also be jeopardized by various cellular processes. During apoptosis, the lamina and nuclear pore complex are targeted by the apoptotic machinery, disturbing the permeability and subsequent breakdown of the NE resulting in destruction of the nucleus [39] and cell death. A peculiar mode of apoptosis is NETosis in which neutrophils voluntarily disrupt their nuclear envelope and plasmatic membrane in response to an infection to release DNA that forms an extracellular mesh with the goal to trap pathogens such as bacteria and signal the immune system to activate the innate immune response [40].

Aging and senescence also cause a broad range of abnormalities in the nuclear morphology that include enlarged nuclei, loss of circularity, appearance of a multi-lobular nucleus, presence of nuclear envelope invaginations, changes in nucleolus, and relaxed heterochromatin [41]. Such abnormalities induced during senescence have been linked to a decrease of Lamin B levels through enhanced autophagy and decrease in RNA stability [42,43,44], compromising NE rigidity. In contrast, aging cells exhibit changes in Lamin A/C localization, with decreased expression in the nucleoplasm and a concomitant accumulation at the nuclear rim, leading to a reduction in heterochromatin-specific tri-methylation of Lys^9^ on histone H3 (Tri-Me-K9H3) [45]. These changes in heterochromatin might compromise the homeostasis forces applied to the NE and therefore disrupt the normal shape. The NPC is also affected in aging cells resulting in compromised nuclear permeability barrier and an accumulation of cytoplasmic tubulin, which is a finding consistent with ‘leakiness’: a loss of nucleo-cytoplasmic compartmentalization [46]. Lamins are also important regulators for telomere maintenance, and telomere shortening during aging could affect lamin organization and composition [47].

### 2.3. Cancer

**Figure 3:**
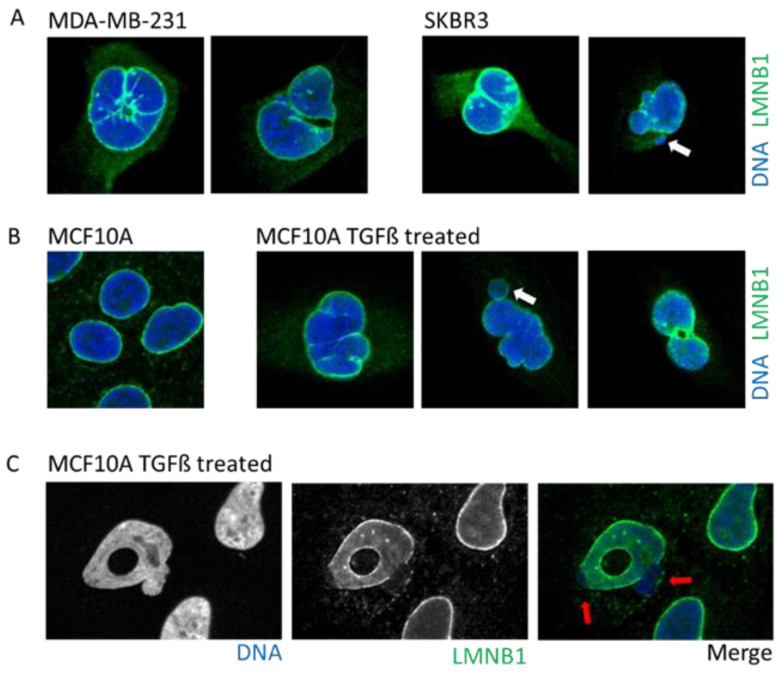
Example of cells with abnormal nuclear envelope. (**A**). MDA-MB-231 and SKBR3 breast cancer cells presenting invaginations, twisted nuclei, and micronuclei (white arrow). (**B**). The breast epithelial cell line MCF10A enters in Epithelial to Mesenchymal Transition upon treatment with TGFß. Upon transformation, cells present nuclear invagination, the presence of micronuclei (white arrow), or donut-shaped nuclei. (**C**). Confocal image of TGFß-treated MCF10A cells highlight nuclear blebbing (red arrow) with a rupture in the Lamin B1 network. Cells are stained for Lamin B1 (LMNB1-green) and DNA (dapi-blue). Adapted from Comaills et al. [10].

Morphological irregularities of the nucleus are not only a characteristic of aging and senescent cells but also a diagnostic factor for tumor cells (Figure 3A) [37]. Nuclear morphology allows the classification of cell states by pathologists [28,29], such as in the case of cervical cancer and the examination of cervical cells using the Papanicolaou smear test. In this test, progression toward cancer due to the infection with the Papilloma virus is characterized by a strident and folded nuclear envelope as well as the presence of micronuclei and multi-lobular nuclei [28,48]. Another example is the pancreatic ductal adenocarcinoma where during progression toward cancer, the nuclear membrane acquires an irregular shape, resulting in an enlarged nucleus and increased nucleus/cytoplasm ratio as well as the presence of macronucleoli [49,50].

Tumors cells can display a myriad of nuclear aberrations such as a loosened NE, twisted nuclei, and invagination, as observed in breast tumor MDA-MB-231 and SKBR3 cells (Figure 3A). Such NE abnormalities can stem from an increase in DNA content, gain in chromosomes and/or ploidy, changes in epigenetic state, and modification in the compaction of chromatin, all increasing the nucleus size. Genetic instability is one of the hallmarks of cancer and is frequently associated with the presence of micronuclei. In some instances, cancer cells may express higher levels of progerin, the immature and farnesylated form of Lamin A, favoring NE defects [51].

### 2.4. Cellular Plasticity and Its Effect on Nuclear Envelope

Cells are known to be subject to cellular plasticity in response to the microenvironment signaling upon injuries and inflammation [52,53]. Those transient changes in cellular identity can have a profound effect. Cellular plasticity entails rapid epigenetic changes with remodeling of the chromatin, as a consequence of repression and/or activation of genomic regions through the modulation of the Lamin-Associated Domains (LAD) among other regions. One outstanding question is whether such alterations modifies the NE composition to adapt to this new cellular state.

One of the most studied cellular plasticity is the transient and reversible epigenetic reprogramming of Epithelial-to-Mesenchymal Transition (EMT) that allows cells to gain migration and invasiveness properties. EMT drives important aspects of embryologic development such as gastrulation, neural crest, and mesectoderm or heart development [54]. Furthermore, EMT seems to be a major player during the metastasis process [54,55] to allow primary tumor cells to invade new environments. EMT can be induced by TGFß, a cytokine detected under an inflamed microenvironment, or release by platelets in the bloodstream. The EMT induces a rapid opening of the chromatin to increase the accessibility of repressed genomic regions as well as enhancers [56]. Interestingly, a study using breast epithelial cell lines revealed that activation of the EMT program decreased protein levels, but not those of RNA, of several nuclear envelope proteins such as lamins, NPC, and nucleolus proteins [10]. An induction of EMT by either TGFß treatment or overexpression of the transcription factor SNAIL prompts profound alterations in the NE morphology that includes blebbing, twisting, invagination, and donut shape nuclei, which is a phenotype associated with decreased farnesylation of Lamin B1 [57] (Figure 3B).

Importantly, a decrease in nuclear envelope proteins leads to failed mitosis and the formation of lagging chromosomes during the anaphase, resulting in the formation of either MN or binucleation in up to 10% of cells [10]. Correlation between the presence of MN and binucleated cells and mesenchymal state was also observed in circulating tumor cells isolated from estrogen receptor-positive breast cancer patients [10,58].

### 2.5. Envelopathies

Envelopathies are rare diseases stemming from mutations in nuclear envelope encoding genes including lamin (i.e., laminopathies), proteins from the inner nuclear membrane, such as emerin or SUN proteins, outer nuclear membranes, such as nesprins and proteins involved in the regulation of NE proteins such as ZMPSTE24 [59]. Envelopathies display a large variety of clinical symptoms including metabolic syndrome, muscular dystrophy, lipodystrophy, neuropathy, and progeria (premature aging), among many others. Lipodystrophies result in an array of metabolic complications as insulin resistance, type 2 diabetes, hypertriglyceridemia, and hepatic steatosis [59,60,61,62,63]. Interestingly, patients with metabolic syndrome also exhibit an unusual high prevalence of laminopathies [64]. To date, nearly 500 mutations have been identified in *LMNA*, which cause a plethora of diseases such as Emery–Dreifuss Muscular Dystrophy (EDMD), dilated cardiomyopathy (DCM), Hutchinson–Gilford Progeria Syndrome (HGPS), Lipodystrophy syndrome, and peripheral neuropathy. The latter highlights the complex genotype–phenotype associations and clinical heterogeneity. Thus, the same variant can lead to different phenotypes, and a similar phenotype can arise from different variants.

Envelopathies clinical heterogeneity is also expressed in overlapping syndromes such as lipodystrophy with myopathy, neuropathy, and/or premature aging stigmata, giving rise to the concept of a multisystem dystrophy syndrome [65].

Similar to the heterogeneity of clinical phenotypes, mutations in different NE protein can also differentially impact NE integrity. For example, cells bearing mutations within the *Lamin A* gene that leads to premature aging HGPS, display an armada of nuclear alterations such as nuclear blebbing, micronuclei, and a honeycomb pattern [66], as well weakened adaptability to external mechanical stress [67]. Importantly, mutations in the *LMNA* gene can result in the loss of proper compartmentalization, leading to transient nuclear envelope disruption (NED) during interphase [68].

## 3. Nuclear Envelope Disruption

The fundamental role of the nuclear envelope is to protect the genome from the damaging effects of the cytoplasm. However, when challenged by either nuclear envelope abnormality or variations in the mechanical force homeostasis, the NE can collapse, impairing proper compartmentalization and exposure to the cytoplasm. Such an event, especially during interphase, when the genome is unfolded and unprotected, can have profound and long-term effects on the genome, including massive genomic instability and the induction of a pro-inflammatory immune response.

### 3.1. Nuclear Envelope Disruption and Repair

**Figure 4:**
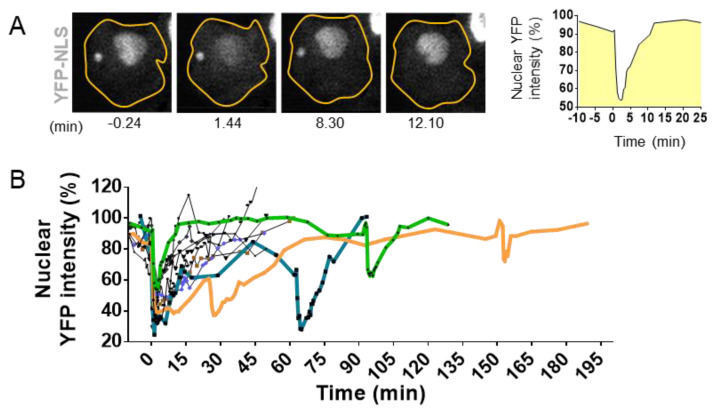
Visualization and quantification of nuclear envelope disruption (NED). (**A**). Time lapse imaging of migrating MCF10A cells treated with TGFß and stably expressing the fluorescent markers YFP linked to the Nuclear Localization Signal (YFP-NLS) [74]. Confocal imaging in YFP channel and bright field allows the visualization of sudden leakage of YFP-NLs into the cytoplasm, showing a loss of proper compartmentalization of the nucleus during 8.30 mn and the loss of compartmentalization of the micronuclei at 12.10 mn of time. (**B**). Graph shows nuclear fluorescence intensity changes upon time in 10 cells with events of interphase NED/nuclear envelope repair observed in TGFß-treated MCF10A cells. Bold colored lines highlight repetitive NED events from the same cell. Adapted from Comaills et al. [10].

The development of high-resolution microscopy tools for the observation of living cells has allowed the detection of NED events during interphase. Using time lapse imaging in cells with a stable expression of fluorescent markers linked to a nuclear localization signal (NLS), it has been possible to observe the proper nuclear compartmentalization [10,35,36,69,70,71] (Figure 4) as well as to quantify the time of NED rupture and repair. The collapse of NE can vary from seconds up to several hours [10,69,72] (Figure 4A,B). Resealing of the nuclear envelope is driven by the Endosomal Sorting Complexes Required for Transport III (ESCRT III) machinery [35,36]. Upon NE rupture, CHMP7–LEMD2 complexes accumulate to the site of rupture and recruit the soluble monomeric ESCRT-III subunits that will polymerize into filaments at the site of rupture to drive membrane sealing [73]. However, in the case of micronuclei, the repair of the NE is impaired [73], and the rupture is sustained until the next mitosis cycle [71].

### 3.2. Causes of Nuclear Envelope Disruption

**Figure 5:**
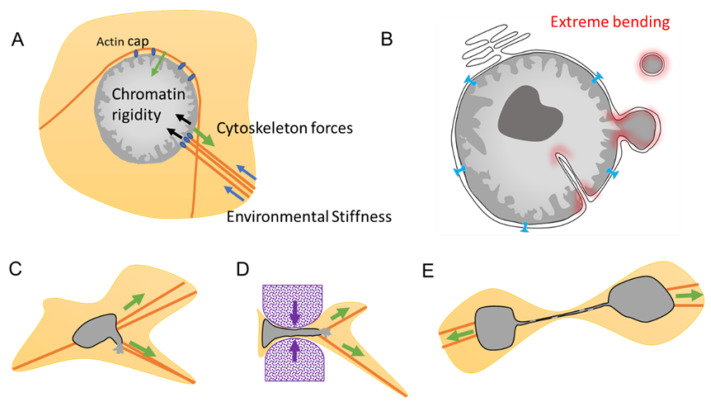
Nuclear envelope (NE) equilibrium and cases of nuclear envelope disruption (NED). (**A**). NE homeostasis between the chromatin rigidity forces (black arrow), cytoskeleton forces (green arrow), and the effect of the environment stiffness (blue arrow). Mechanosensitivity of the NE is done through the connection of the cytoskeleton by the LINC complex (blue ovals). (**B**). Example of nucleus with several NE abnormalities leading to extreme NE curvature at invagination, blebbing, or on the NE of the micronuclei that might be involved in NE fragility (highlighted in red). (**C**). Cells under migration have strong cytoskeleton attraction that can lead to local tension and the formation of nuclear envelope disruption. (**D**). Cells in migration through tiny constrictions also endure new stiffness forces and will deform NE locally to ensure the nuclear passage and create new NE tension and extreme NE bending, leading to NED. (**E**). Two daughter cells experiencing telomere fusion have their nucleus connected by a chromatin bridge and sharing the same NE. The need of extra NE to cover the extended NE surface leads to NE weakness and break.

The NE homeostasis is achieved through a balance between different mechanical forces from within and from outside the nuclei (Figure 5A). From within, forces are determined by the chromatin rigidity that vary upon the chromatin state and compaction of the heterochromatin. Drastic changes in heterochromatin density can occur during cellular plasticity such as during EMT [56,75] or during aging [45]. Interestingly, the chromatin is also mechanoresponsive, and chromatin can alter its own mechanical state to maintain genome integrity in response to deformation [76].

From the outside, the nucleus possesses mechanosensitive properties via its connection with the cytoskeleton. As a result, gene regulation can drastically change depending on the stiffness of the environment and can result in cellular differentiation [2]. The Linker of Nucleoskeleton and Cytoskeleton (LINC) complex bridges the nucleus to the cytoskeleton (Figure 1C and Figure 5A) and is the major force-transmitting sensor. LINC serves as a mechanosensor, translating mechanical cues, which include physical forces compression of the actin cap [77], shear stress, and alterations in extracellular matrix stiffness, into biochemical signals, thus allowing cells to adapt to their physical environment [78]. Cells also monitor their own shape and develop an active contractile response when the nucleus deformed below a specific threshold. Transition in the mechanical state of the NE induces calcium release, activating the calcium-dependent phospholipase cPLA2 and downstream myosin II, causing cells to move with the goal to rescue nuclei from a constraint area [79].

Maintenance of the NE equilibrium is conveyed by a balance between the various forces imposed upon it, which can be disrupted under certain conditions leading to transient NED. For example, cancer leads to improper NE regulation characterized by NE blebbing, micronuclei, and invagination. Such formation involves an extreme bending of the NE where the mechanical properties of the membranes may be important, weakening the integrity of the NE and leading to the plausible cause of NE collapse (Figure 5B).

#### 3.2.1. Alterations in Expression of Lamins

NED can be provoked as a result of decreased lamin expression as observed during epigenetic reconversion of EMT where all the lamins are downregulated [10], or by experimentally targeting type B lamins using shRNA constructs [69,72]. Alterations in lamin expression affect the rigidity and flexibility of the nucleus and disrupt the NE force equilibrium that can end up in transient collapse. As such, in laminopathy, cells with mutations in the *LMNA* gene have a weaker nuclear envelope and are more susceptible to ruptures [80]. Indeed, 29% of fibroblasts derived from progeria patients display nuclear envelope collapse [68].

#### 3.2.2. Migration and Invasion

During migration, the cytoskeleton imposes considerable traction forces, resulting in strenuous nucleus deformation. The swift reorganization of the cytoskeleton in response to cues may surpass the capacity of the NE to adapt, resulting in the formation of blebbing that can ultimately lead to NED, as observed both in vitro and in vivo during migration [10,35,36] (Figure 5C). Passage through restrained spaces such as during extravasation and passage through capillary increases the local environmental stiffness force, leading to nucleus compression and to extreme NE deformation and curvation that may result in NED [35,36,81]. The ratio of Lamin A/B determines NE flexibility and rigidity, and thus, its capacity to proceed, or not, through restrained spaces [1]. For example, NE blebbing is characterized by reduced Lamin B1 levels, suggesting that Lamin B filaments are overly stiff and unable to bend on high-curvature nuclear membranes, which is a phenomenon that can be explained by Lamin B high affinity for the NE lipid layer due to their farnesylation and attachment to Lamin B Receptor [81] (Figure 3C).

#### 3.2.3. Micronuclei: NE Extreme Curvation and Improper NE Composition

MN are small nuclei containing either a full chromosome that lagged during mitosis or a fragment thereof missing a proper centromere. Several studies have shown that the NE of MN are susceptible to rupture without repair capability [71] (Figure 4A). MN disruption could be due to issues in assembly and composition, as essential NE proteins are lacking [71,82], and 40% of MNs fail to import NLS tagged protein [82]. Interestingly, MN are characterized by lower levels of Lamin B, and the presence of functional Lamin B partly correlates with micronuclei size, suggesting that the right assembly of NE might be sensitive to membrane curvature [83]. A recent study [73] also shows that MN lacks the capacity to repair the NE after a collapse due to the lack in restricting CHMP7–LEMD2 complexes to the site of rupture, resulting in an unrestrained activation of ESCRT-III across the surface of their inner membrane. Rather than repairing the ruptured micronuclei, the hyperaccumulation of ESCRT-III drives dramatic membrane distortion and causes DNA torsional stress, the formation of single-stranded DNA, and chromosome damage [73].

#### 3.2.4. Telomere Fusion

Telomere fusion is characterized by the fusion of two telomeric ends between two chromosomes, called end-to-end fusion, which is a phenomenon often observed in cancer and aging [84]. This telomere crisis leads to dicentric chromosomes that invariably persist through mitosis and form long chromatin bridges that connect daughter cells well into the next G1 phase [85] (Figure 5E and Figure 7). These chromatin bridges are surrounded by a contiguous nuclear envelope that increase the overall NE surface. Stretching of the NE is often associated with rupture of the nuclear envelope of the connected nuclei [70] (Figure 5E). This NED is transient and last around 2 min [70]. Interestingly, the NE protein composition in this chromatin bridge appears to have the same defects than some MN that were trapped in the spindle during the NE formation. By being trapped in the middle of the spindle, the assembly of the NE seems compromised and appears to lack several essential NE proteins such as NPC, affecting the NE maintenance [82].

#### 3.2.5. ATR, RB, and P53 Loss

The modulation of proteins levels involved in genomic stability can lead to NE deformation and rupture. ATR is a serine/threonine protein kinase that activates checkpoint signaling upon genotoxic or replication stresses, thereby acting as a DNA damage sensor. Activated ATR performs multiple cellular functions to maintain genomic integrity and the prevention of replication and mitotic catastrophe [86]. However, ATR is also known to have other functions such as maintaining NE integrity in response to mechanical stress [87]. Mutations in the *ATR* gene as well as shRNA-mediated silencing compromise NE integrity and are associated with NE blebbing [87] and invaginations as well as transient NED [88]. The NE lipid composition is altered in ATR-silenced cells [87], substantiating the roles of ATR in the NE adaptability and regulation, as well as in heterochromatin compaction, affecting the nuclear stiffness. In vitro studies have also shown that cells depleted for either retinoblastoma protein (Rb) or the tumor suppressor P53 exhibit increased incidence of NE rupture [89]. Such a phenotype is not associated with greater mobility nor changes in NE protein composition, but it seems to be linked with genome reorganization and increased nuclei size [89]. These examples demonstrate the complex relationship between genome organization, genome maintenance, and NE integrity.

#### 3.2.6. Heterochromatin Modulation

Chromatin compaction profoundly affects nucleus stiffness (Figure 5A). Certain cellular states or transient epigenetic transformation can induce a complete chromatin landscape reorganization such as during EMT [56,75] or during aging [45]. These changes modify homeostasis, which can weaken the NE and develop NED [10]. Another evidence of the role of chromatin compaction on NE homeostasis is the modulation of the nucleosome binding protein HMGN5. Study in vitro and in vivo have shown that the overexpression of HMGN5 leads to enlarged nuclei and NED [90]. The effect of HMGM5 overexpression in the NE integrity was more obvious in contractile tissues due to extensive cytoskeleton forces. This study confirms that heterochromatin provides mechanical stability to the nucleus. Then cellular plasticity might have more profound effects than expected and could drive NE fragility, deformation and eventual collapse.

#### 3.2.7. Virus Infection

Many DNA as well as several RNA viruses hijack the host replicative system in order to propagate and as such have developed various mechanisms to shuttle in and out of the nucleus, either by usurping cellular transport pathways through the nuclear pore complex or translocating directly through the NE [91,92]. Interestingly, some viruses use non-invasive mechanisms in order to secure proper and efficiency replication of the particle while escaping the activation of cytoplasmic DNA sensor pathways. In contrast, other viruses induce the nuclear and membrane breakdown later in infection in order to release mature viral particles, as in the case of adenoviruses, papillomaviruses, and polyomaviruses [91]. As a result of their smaller size, non-enveloped viruses can provoke transient NED as observed during early infection of the mouse parvovirus minute virus (MVM) [92,93,94]. The early infection of human papillomavirus type 16 requires breakdown of the NE to access the nucleus, and it is believed they enter during the mitotic NED. However, it is possible they use other mechanisms such as transient NED. It is also important to note that many viruses destabilize the cell cycle [95], causing mitotic defect that can result indirectly in NED. For example, Hepatitis B virus X protein affects S phase progression, leading to chromosome segregation defects [96]. Lagging chromosomes will generate MN and subsequently give rise to NED and ultimately genomic instability and cancer development. Such a process could potentially be implicated in the early stages of liver cancer.

## 4. Cytoplasmic DNA Induces Inflammation by cGAS/STING Pathway

**Figure 6:**
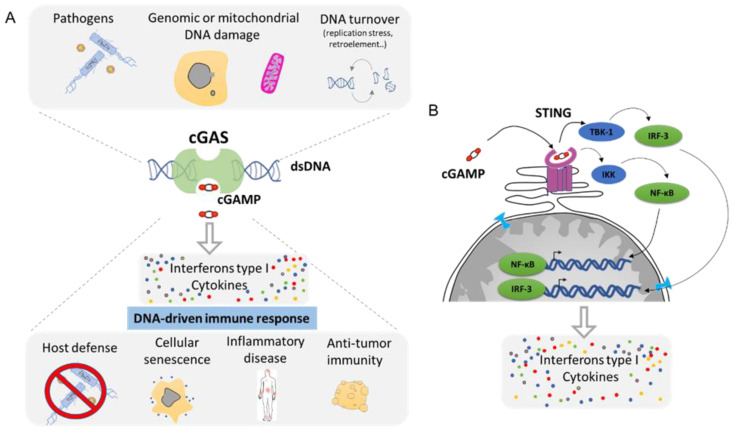
The cGAS–cGAMP–STING signaling pathway: a universal sensor for double-strand DNA (dsDNA). (**A**). cGAS function is to sense DNA from the cytoplasm as result of infection, genomic, or mitochondrial instability. cGAS activation through the generation of cGAMP will drive the activation of the innate immune response and leads to the secretion of Interferon type 1 as well as a cocktail of cytokines. (**B**). Molecular mechanism of cGAS/STING pathway. cGAMP is the ligand for the STING receptor (shown in pink) and results in the activation of the transcription regulator factors NF-κβ and IRF-3 that drive the secretion of several pro-inflammatory molecules (Adapted from Ablasser et al. [100] and Motwani et al. [97]).

Any DNA within the cytoplasmic compartment is sensed as foreign DNA, mimicking a viral or bacterial infection, resulting in the activation of DNA recognition pathways by the cell in order to neutralize the invader. In mammalian cells, the three major DNA-sensing receptors that drive immune responses to foreign DNA are the Toll-like receptor 9 (TLR9), the absent in melanoma 2 (AIM2), and the cyclic GMP–AMP synthase (cGAS) [97]. These DNA-sensing receptors are mostly expressed in blood and immune cells with the exception of the cGAS pathway that can be induced in several cell types. cGAS is an intracellular enzyme that binds to double-stranded DNA (dsDNA) and initiates a tightly regulated signaling cascade to induce the expression of inflammatory genes. The classical role of cGAS is to detect DNA from pathogens, such as bacteria and virus, and to activate the innate immune pathway (Figure 6A). However, the origin of DNA in the cytoplasm can be diverse, and cGAS can be activated by DNA from its own cell. The outcome of genomic instability is ultimately linked to the release of DNA in the cytoplasm such as during DNA damage or NED from MN and nucleus. Mitochondria can also release part of the genome during mitochondrial stress or during apoptosis [98]. DNA produced during the resolution of replication stress or retroelements such as retrotransposons are also a source of aberrant cytoplasmic DNA (Figure 6A). This DNA is usually degraded by the TREX1 protein, which is a DNA exonuclease that clears normal endogenous cytosolic DNA to prevent aberrant stimulation of the cGAS pathway [99].

Mechanistically, cGAS activation by cytosolic DNA leads to the generation of cyclic GMP–AMP (cGAMP), which binds to the receptor STImulator of INterferon Genes (STING). Activation of STING results in the activation of TANK-binding kinase 1 (TBK1) and in the dimerization and nuclear translocation of the interferon regulatory transcription factor 3 (IRF3), resulting in the transcription of genes encoding type I interferons (reviewed in [100,101]) (Figure 6B). DNA sensing through the cGAS–STING pathway also results in the activation of the nuclear factor NF-κB through IKK and leads to the transcription of pro-inflammatory cytokines such as IL-6 and tumor necrosis factor (TNF) [102]. This cocktail of signal attracts, alerts, and activates the immune system of a potential danger. Then, the cGAS pathway is involved in several functions such as host defense in response to bacterial or viral infection. It supports the natural antitumor activity by facilitating the recognition of cellular damage and in promoting cellular senescence [100,103,104] (Figure 6A). However, the cGAS/STING pathway plays also a crucial role in many inflammation-related diseases such as cardiovascular disease, neurodegenerative disease, inflammatory bowel disease, metabolic syndrome such as diabetes, fibrosis, lupus, arthritis, and psoriasis [100,105,106] (Figure 6A).

## 5. Chromosomal Instability Associated to NED

Nuclear envelope disruption destroys the barrier between cytoplasmic components and the genomic DNA, which may have devastating consequences on the genome pending time of NE repair. In extreme cases, mitochondria have been trapped within the nucleus during transient NED [69]. Several studies have demonstrated that nuclear envelope collapse can have a major role on tumor evolution and in the creation of genomic diversity. NED can lead to massive DNA damage as well as diverse complex chromosomal rearrangements, such as chromothripsis [107] or common deletion/insertion events [108].

### 5.1. Diverse Spectrum of Chromosomal Rearrangements Derived from MN Studies

**Figure 7:**
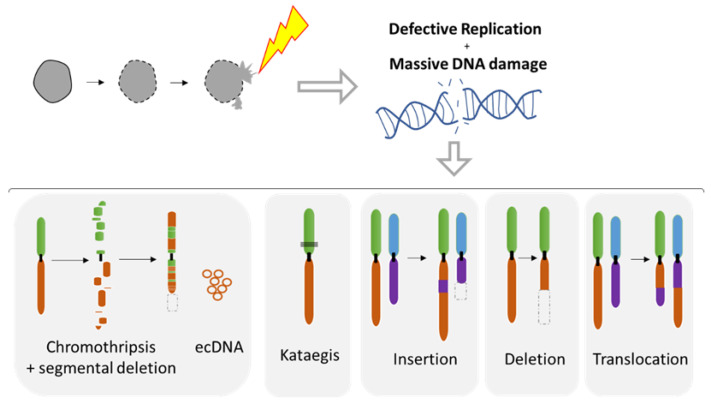
Chromosomal instability resulting from nuclear envelope disruption of micronuclei (MN). Defects in nuclear envelope of the micronuclei lead to it collapsing, resulting in defective replication and massive DNA damage. MN collapse is associated with a broad range of chromosomal rearrangements such as chromothripsis, the hyper mutated pattern kataegis, as well as the insertion, deletion, and translocation events. Circular chromosomes (ecDNA) are often associated with chromothripsis.

The MN shelters either an intact chromosome or a fragment thereof as a result of aberrant mitosis. MN formation can be induced in vitro using either mitotic spindle inhibitors (e.g., Nocodazole release) [71,107,109] or by impairing the kinetochore attachment on the Y chromosome [108], thus allowing the study of chromosome instability. Indeed, MN are a site of intensive DNA damage as observed with the DNA damage marker gamma H2AX [71,109,110]. As the nuclear envelope of MN tends to disrupt and is unable to repair [71], this process can lead to massive DNA damage, ultimately producing chromosome fragmentation and pulverization [71,107,110,111] (Figure 7). Deficiency of MN nuclear envelope composition [82] can also be responsible for defective replication in both intact and disrupted MN [109,110]. The lack of proper replication leads to a desynchronization during the mitosis, resulting in massive DNA breaks during metaphase [109,110] and leading to chromosome pulverization. Pieces of pulverized chromosome can randomly reassemble, leading to chromothripsis, which is characterized by up to one hundred chromosomal rearrangements that occur all at once (Figure 7). MN generates chromothripsis at remarkably high rates [83,107,112,113,114]. It is also important to note that chromothripsis is associated with segmental deletion and the production of circular extrachromosomal DNA (ecDNA) amplification (also known as double minutes) [108,115]. ecDNAs are found in nearly half of cancers and contribute to oncogene amplification as well as to tumor genetic heterogeneity [116]. MNs can also result in other types of chromosomal rearrangements such as deletion, insertion, or translocation events as well as kataegis, which is a pattern of localized hypermutations [108].

### 5.2. Telomere Fusion and Chromosomal Bridge

**Figure 8:**
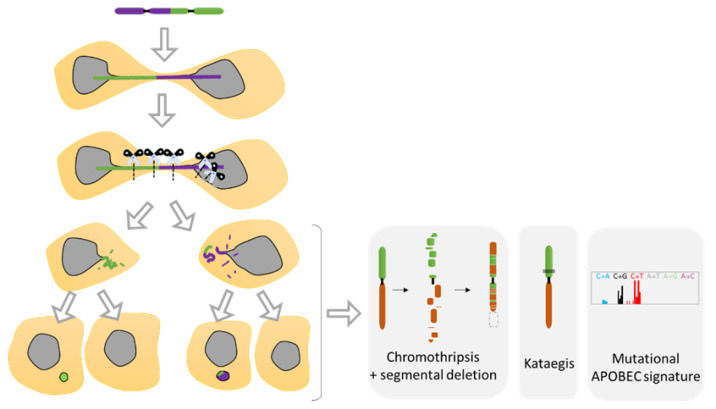
Chromosomal instability resulting from nuclear envelope disruption (NED) during telomere fusion. Dicentric chromosome under mitosis ends up in two daughter cells connected by a chromatin bridge. Studies have shown that the dicentric chromosome will break in response to NED and the attack of TREX1 proteins as well as due to extensive mechanical forces from the cytoskeleton. The resulting breaks will generate diverse structural variants as the formation of chromothripsis, kataegis as well as a located mutagenesis APOBEC signature. Furthermore, defective replication on the broken bridge of the chromosome scales up the genomic instability suffered by the cells that will progress in the formation of micronuclei in the next mitosis, increasing considerably the genomic instability (Adapted from Umbreit et al. [110] and Maciejowski et al. [70]).

Recent studies using the model of telomere fusion have revealed massive DNA damage and reorganization on the fused chromosomes as a result of NED [70,117] (Figure 5E and Figure 8). The sequencing of clones derived from telomere fusion has shown events of chromothripsis, as well as kataegis and displayed clusters of genomic rearrangements affecting one or more chromosomes (Figure 8). These rearrangements exhibited the hallmarks of chromothripsis, including spatial clustering, randomness of fragment orientation, and oscillating copy number states [70]. Mechanistic studies have highlighted TREX1 as an important contributor of DNA breaks and chromothripsis [70,117]. TREX1 is a powerful DNA exonuclease that degrades both double- and single-strand DNA from the 3′-terminus and that is required for clearing cytosolic DNA to prevent aberrant inflammation and autoimmunity [99]. In addition, APOBEC3 is likely involved in the generation of the hyper mutated pattern kataegis observed in this model [117] (Figure 8). APOBEC3 functions as DNA mutator participating in the innate immune system. It is a DNA deaminase that acts as an inhibitor of retrovirus replication and retrotransposon mobility and target cytoplasmic DNA. During nuclear envelope collapse, APOBEC and genomic DNA come into contact, inducing aberrant APOBEC-mediated mutagenesis. The primary biochemical reaction induced by the APOBEC family of proteins is cytosine to uracil (C-to-U) deamination. However, cytosine to guanine (C-to-G) and cytosine to thymine (C-to-T) transitions, and other mutations can be induced by these enzymes [118].

A recent study demonstrates that mechanical force provoked by the accumulation of contractile myosin II can also trigger chromosome bridge breakage rather than the effect of TREX1 [110] and NED. Both events might appear to be non-mutually exclusive and complementary. Furthermore, the study elegantly demonstrates the presence of defective DNA replication of bridge DNA, which can generate complex rearrangements such as “Tandem Short Template (TST) jumps” [110]. Moreover, the under-replicated bridge chromosomes mis-segregate with high frequency and form micronuclei in the following cell cycle, which can generate additional cycles of bridging, micronucleation, and chromothripsis [110,119].

### 5.3. DNA Damage Arising from NED of the Nucleus

DNA damage from NED was primarily studied in the case of micronuclei [71,109] where the NE rupture is permanent, leaving the chromosomal contents therein completely exposed to the surrounding environment and leading to massive DNA damage. In the case of chromatin bridge, NED is transient, lasting less than 2 min and potentially recurrent. As such, it is difficult to assess the contribution of these transient NED on genomic instability [70,110,117]. In contrast, NED observed under certain diseases such as envelopathies or during migration and invasion can last up to 1 h (Figure 4B). DNA marker γH2AX or 53BP1 foci show important DNA breaks in the constricted nuclei during migration through restrained areas [35,36,120], confirming the generation of DNA double-strand breaks in the genome during transient NE collapse. The incorporation of organelle such as mitochondria has also been observed after NE repair, questioning the fate and consequence of such an intruder on nuclear dynamics [69]. Interestingly, 1% of rat cardiomyocytes, a cell type that endures persistent mechanostress, present mitochondria in their nucleus, giving more importance to this phenomenon [121].

Both NED and NE blebbing involve a transient chromatin delocalization from DNA leaking to the cytoplasm during the rupture, which is rapidly reintegrated in the nucleus during repair. Whether this reintegration is faithful to the original chromosomal territory remains to be assessed. In the situation of double-strand DNA breaks, the broken ends may no longer be in close proximity. The repair and ligation of two ends can result in chromosomal translocation events. Importantly, a recent study using the CRISPR–Cas9 genome editing revealed that a single double-strand DNA break causes up to a 20-fold increase in the formation of micronuclei and/or chromosome bridges [114]. As such, the damage cause by NED can be amplified into far more extensive genetic alterations in subsequent mitosis.

The relationship between DNA damage response and NE is complex, and it is known that several NE proteins play important functions during DNA damage repair, such as the proteins from the LINC complex [122,123] or the Lamin-Associated Protein 1 (LAP1) [124]. Then, the disruption of NE integrity also compromises the proper DNA repair.

## 6. Clinical Consequences of Nuclear Envelope Rupture

The release of genomic DNA into the cytoplasm leads to major consequences with diverse clinical outcomes: the creation of chromosomal instability and the induction of pro-inflammatory pathways. Recent technological advances in sequencing tools as well as imaging have shifted interest in the role of NE fragility as the origin of some congenital diseases, in the creation of cancer, and to certain inflammatory syndromes. The role of NED and activation of the innate immune response through cGAS could be in fact linked to much more diseases than expected.

### 6.1. Envelopathies and Inflammation

Nuclear envelope abnormalities due to mutation in NE proteins are well described and are associated to a myriad of clinical phenotypes, ranging from cardiac and skeletal myopathies to partial lipodystrophy, peripheral neuropathy, and premature aging [125]. The recent discovery of NED during interphase observed with the use of time lapse imaging has allowed a better understanding of the role of certain mutations on NE integrity, highlighting the implication of NED, genomic instability and activation of the innate immune response in the establishment of tissue degeneration. Most of envelopathies are tissue-specific, affecting the skeletal muscle, heart, peripheral nerves, bone, or adipose tissue, and they are caused by mutations in ubiquitously expressed proteins, as are the proteins from the NE. Then, the disruption of their function may be limited by epigenetic and specific chromatin compaction, or in response to specific stiffness of their environment, such muscle cells that are constantly confronted to mechanostress. However, the link between NED and local inflammation could explain the diversity of pathology, and the role of cGAS/STING pathway activity during NED could be in fact largely underestimated.

#### 6.1.1. Emery–Dreifuss Muscular Dystrophy: Cell Death Due to Increase DNA Damage

The Emery–Dreifuss muscular dystrophy (EDMD) is a rare disease caused by mutations in nuclear envelope proteins such as Lamin A, Emerin, or Nesprin 1 and 2. The clinical symptoms are skeletal muscle wasting, joint contractures, cardiomyopathy, and congenital muscular dystrophy. Using several mice models and patients’ samples with mutated *Lamin A* [80] or in *SYNE-1* [126] (coding for the Nesprin 1 protein) genes, researchers observed the presence of NED during muscular contraction. Muscle cells bearing one of the EDMD mutations display increased DNA damage and increased apoptosis. Importantly, NED also led to activation of the cGAS pathway [80], which might play a major role in the development of the disease.

#### 6.1.2. Progeria: DNA Damage, NED, and cGAS Activation

Hutchinson–Gilford progeria (HGPS) arises from single point mutations within the *Lamin A* gene, leading to its permanent farnesylation, also called progerin, and it leads to premature aging, among other symptoms. Patients die from complications of atherosclerosis, such as heart attack or stroke in their early teens. Atherosclerosis is a chronic inflammatory disease of the vessel wall that is largely driven by an innate immune response [127], and it seems to be driven by the cGAS/STING pathway [128]. A study of cells from progeria patients has shown NE fragility that tends to disrupt [68]. In addition, progerin expression renders smooth muscle cells more susceptible to cell death in response to mechanical stress, which is likely due to nuclear envelope disruption [129]. Other studies have shown that progerin expression activates the cGAS/STING/IFN pathway [130,131,132] and suggest that this activation is due to either replication stress or to oxidative stress. However, the role of NED in this pathway activation cannot be excluded.

#### 6.1.3. Metabolic Syndrome: A Common Symptom in Envelopathies

Some common clinical symptoms of envelopathies patients are the lipodystrophy and lipoatrophic diabetes (Table 1). However, the link between metabolic syndrome and nuclear envelope dysfunction is not yet understood. Lipodystrophies are characterized by near-total loss of body fat. In view to compensate for the lack of adipocyte tissues, the fat is stocked in ectopic fat stores, particularly certain subcutaneous depots but also within and around the skeletal muscle, heart, liver, pancreas, and kidneys, leading to deregulation in metabolic homeostasis [133]. The syndrome can result in an array of metabolic complications such as insulin resistance, type 2 diabetes, hypertriglyceridemia, and hepatic steatosis. Interestingly, lipodystrophy is often associated with autoimmune disorders including lupus, dermatomyositis, Celiac disease, pernicious anemia, and vasculitis [133,134], highlighting the role of immunity in this disease. Whether NED-mediated cGAS activation is a major factor in the development of this clinical symptom is an open question.

In favor of such a premise, mutations in lamin and lamin-associated genes are known to induce seven syndromes associated with lipodystrophy and lipoatrophic diabetes (Table 1). Lamins are known to regulate gene expression at the epigenetic level through their Lamina-Associated Domains (LADs). Consequently, mutations in lamin genes may impact the regulation of metabolic genes in a tissue-specific manner as each tissue possesses a distinct epigenetic statue. Nonetheless, a general hallmark of dystrophy is the degeneration and death of tissues, suggesting that additional and more mechanisms are also involved. The link between NED, cGAS activation, and lipodystrophy is not yet known, but in the case of some *Lamin A* mutations such as HGPS and EDMD, it was demonstrated that the NE can collapse [68,80], highlighting a probable role of NE fragility and plausible NED and cGAS activation in the development of such clinical outcomes. Interestingly, laminopathies seem to be under-diagnosed, as a study analyzing metabolic syndrome reported that 10% of patients presented abnormal nuclear envelope and 3.6% possessed a mutation in the *Lamin A (LMNA)* gene [64].

Interestingly, *BSCL2* and *AGPAT2* gene mutations induce Berardinelli–Seip congenital lipodystrophy type 2, which is the most severe form of human lipodystrophy. BSCL2 is a reticulum endoplasmic protein involved in the regulation of lipid droplets by facilitating continuous triglyceride transfer [135]. BSCL2 is also a key factor for nuclear lipid droplet generation and lipid homeostasis [136]. Indeed, BSCL2 connects the lipid layer from the inner NE and the nuclear lipid droplet, and it might affect the inner nuclear membrane maintenance [136]. AGPAT2 converts lysophosphatidic acid to phosphatidic acid (PA), which is the second step in de novo phospholipid biosynthesis. Nevertheless, PA are substrates for enzymes producing lipids that are involved in fission or fusion, contributing to membrane rearrangements by generating negative membrane curvature [137]. Then, both proteins are important players in the maintenance of the lipid bilayer that might be involved in NE adaptability to external forces and could present NE fragility in certain context as in adipocytes.

#### 6.1.4. Neuropathies, Nuclear Envelope, and Inflammation

Mutation in lamin genes can also cause neuropathies [138,139,140,141]. Other genetic diseases that causes neurological lesion are the tauopathies, which are neurodegenerative disorders characterized by the deposition of abnormal tau protein in the brain. Tau is a microtubule-associated protein expressed in neurons that are involved in neurodegenerative diseases, including Alzheimer disease (AD), frontotemporal dementia with parkinsonism-17, Pick disease, progressive supranuclear palsy, and corticobasal degeneration [142]. These diseases indirectly involve the NE dysfunction, as the abnormal localization of Tau generates the production of nuclear envelope invaginations and is associated with lamin dysfunction [143]. Whether such NE deformations are linked with NED is still not fully known, but it was recently described that the nucleocytoplasmic transport that is compromised is a model of frontotemporal dementia that could be in fact the result of NED [144].

Impairment of nucleocytoplasmic transport has also recently emerged as a central disease mechanism in amyotrophic lateral sclerosis and frontotemporal dementia due to hexanucleotide expansions in the *C9ORF72* gene [145] and in Huntington’s disease [146,147]. Expression of those repeats leads to morphological abnormalities in the architecture of the nuclear envelope. It is not clear if the impairment of nucleocytoplasmic transport is due to disruption of nuclear import or due to NE collapse.

Interestingly, chronic activation of an innate immune response in the central nervous system is frequently associated with neuronal damage [148]. Recently, cGAS/STING activation [149,150,151] was discovered to be involved in neurological diseases as multiple sclerosis [152] or Huntington’s disease [153]. The release of genomic DNA into the cytoplasm due to NE weakness and subsequent activation of the cGAS/STING pathway could be then a major driver in the development of neuroinflammation, leading to degeneration of neurons.

#### 6.1.5. Congenital Disease Due to Complex Chromosomal Rearrangement

Most human embryos are aneuploid and do not develop to term, making aneuploidy in embryos a leading cause of miscarriages and infertility [154]. Aneuploidy frequently arises during the early mitotic divisions of the embryo. During the fecundation, the parental genomes fuse and cluster to form the embryo. However, clustering often fails, leading to chromosome segregation errors and micronuclei, which are incompatible with healthy embryo development [155]. Recent advances in sequencing methodology have allowed the detection and the description of complex structural variations inside the genome of patients with developmental disease, as well as in phenotypically normal individuals [156]. Complex chromosomal rearrangements as chromothripsis are detected in developmentally delayed children but also in mothers suffering spontaneous abortions [156,157]. Carriers of chromosomes with chromosomal complex structural reorganization as chromothripsis cannot pair their chromosome with their partners, leading to infertility. The MN formed during the failed clustering of parental genome during the fecundation is hypothetically the origin of those atopic genome.

#### 6.1.6. Cancer: Origin, Evolution, and Survival

The genomic instability linked with NED can have profound effects on cell evolution. These genomic insults not only increase the pool of genetic diverse cells but also events that can progress into cancers. Recent advances in sequencing have allowed the observation that chromothripsis and other complex chromosomal rearrangements are early events leading to tumorigenesis in pancreatic adenocarcinoma [158], multiple myeloma [159], or breast cancers [160], among many others [161,162]. Genetic insults associated with NED contribute to all steps in cancer, from the origin to the progression and establishment of drug resistance. They are indispensable in the generation of genomic heterogeneity.

Chromothripsis accounts for a substantial proportion of human cancers, with a general prevalence of 49% and up to 80% in breast cancers [162]. Forty percent of tumors with chromothripsis harbor only one chromosome but might have more complex patterns with at least five chromosomes affected in 61% of osteosarcomas [161]. Importantly, polyploidy tumors have 1.5 times more probability to generate an event of chromothripsis [161], which is in line with the genetic instability of whole genome doubled cells and increase of MN formation. Circular extrachromosomal DNA or ecDNA, associated with events of chromothripsis, are found in 40% of cancer cells [116] and are primordial in tumor evolution.

The activation of the cGAS/STING pathway in response to NED is an important anti-tumorigenic mechanism via activating the immune surveillance to mediate tumor clearance (Figure 6). It is likely that during the first step of tumorigenesis, the cGAS pathway plays a primordial role in inducing an immune attack or by promoting intrinsic senescence [163]. Nevertheless, mounting evidence suggests that depending on the context, the cGAS/STING pathway can have tumor and metastasis-promoting functions [163]. It was shown recently that metastatic cells harboring high chromosome instability could engage the STING-dependent non-canonical NF-κB pathway as well as suppress anti-viral type 1 IFN production, thereby activating inflammatory responses that favor invasion and metastasis [164]. Metastatic cells can also communicate with their microenvironment through cGAMP signaling. In metastatic human breast tumors, brain metastatic cancer cells converse with adjacent astrocytes through cGAMP generated by the tumor. cGAMP is exported to astrocytes via gap junctions, which in turn activate the STING pathway and initiate the release of inflammatory cytokines, favoring the brain metastases survival [165]. Moreover, STING chronic activation could induce paradoxically an immune-suppressive environment [163]. Cancer cells treated with STING agonist markedly increased PD-L1 expression and pro-inflammatory cytokines [166]. Cancer cells expressing PD-L1 support the evasion of T cell immune surveillance by blocking T cell killing [163]. Although the precise mechanism remains to be elucidated, it is tempting to speculate that RelA/NF-κB signaling stabilizing the PD-L1 protein could be involved in this paradox [167].

## 7. Concluding Remarks

Over the past 10 years, the research on NE biology has revealed unexpected consequences of its deregulation. The discovery of NED and its role in activation of the cGAS/STING pathway as well as its role in creating chromosomal instability has stirred this field. The NE composition is highly malleable and adapts according to epigenetic changes and mechano-stress. However, under certain conditions and due to some genetic predispositions, the NE can collapse. Naked DNA is sensed as a danger by intra-cellular DNA recognition pathways, and in particular, the cGAS/STING pathway as well as by TREX1 or APOBEC. Cells activate the immune response and attack their own DNA, creating massive DNA damage that could generate complex chromosomal rearrangement as chromothripsis, ecDNA, or Kataegis, leading to tumorigenesis. This defense mechanism has major impact leading to tissue degeneration, as in the case of some laminopathy and in neurodegenerative diseases as well as to induce local inflammation that can drive to autoimmune disease. The role of cGAS/STING signaling in immune and autoimmune diseases seems to be a major driver, and there is an increased interest in targeting this pathway. Several molecules and strategies to disrupt this pathway are being delineated and evaluated in preclinical models [105,168] and will soon enter clinical trials. Such discoveries could be translated to several NE associated disease such as laminopathy and also be used in the cancer field in an attempt to block the growth of metastasis.

The genomic instability driven by NED leads to considerable diverse and complex genetic variability. The use of sophisticated sequencing tools has revealed a more important penetrance of complex chromosomic architecture in disease than initially expected. Those sequencing tools might help to diagnose and to understand some rare and orphan genetic syndrome. In the case of cancer, not only those complex chromosomic rearrangement events can promote tumorigenesis, as it was shown to be an early driver event in the establishment of tumors, but it is also an important factor in the establishment of genetic heterogeneity, supporting the creation of drug-resistant clones. To conclude, the understanding of NE dynamic is indispensable in order to understand a myriad of diseases with a broad range of clinical symptoms. The interplay between inflammation, genomic instability, and NE fragility is fascinating, and harnessing its secrets may open new avenues in the development of innovative therapeutic strategies.

## Figures and Tables

**Table 1:** Syndromes associated with lipodystrophy, which is a common pathology in envelopathy patients. Online Mendelian Inheritance in Man (*OMIM*) ID is given to refer to the catalogue of genetic disorder.

Mutated Gene	Name Syndrome	Clinical Pathology	OMIM ID
*Lamin B2*	Barraquer–Simons syndrome (APL)	Lipodystrophy	608709
*Lamin A/C*	FPLD2, LDHCP, MADA, HGPS, WRN	Lipoatrophic diabetes/Lipodystrophy	151660, 608056, 248370, 176670, 277700
*ZMPSTE24*	MADB	Lipodystrophy	608612
*AGPAT2* or *BSCL2*	Berardinelli–Seip congenital lipodystrophy type 2	Lipodystrophy/Insulin resistance	603100, 269700, 615924
